# M-SAKHI – Mobile health solutions to help community providers promote maternal and infant nutrition and health: a description of development of the Program Impact Pathway using Theory of Change

**DOI:** 10.1017/S1368980024001265

**Published:** 2024-10-22

**Authors:** Archana B Patel, Priyanka N Kuhite, Samreen Sadaf Khan, Amrita Puranik, Ashraful Alam, Michael J Dibley

**Affiliations:** 1 Lata Medical Research Foundation, Nagpur, India; 2 Datta Meghe Institute of Higher Education & Research (Deemed to be University), Sawangi, India; 3 Sydney School of Public Health, University of Sydney, Sydney, NSW, Australia

**Keywords:** M-SAKHI, Program Impact Pathway, Theory of Change, Process Evaluation, mHealth, Behaviour Change Communication intervention, Impact evaluation

## Abstract

**Objective:**

Behaviour Change Communication (BCC) intervention programmes often lack documentation of successful processes. This manuscript aims to describe the development of Program Impact Pathway (PIP) using Theory of Change (ToC) approach for a mHealth BCC intervention titled ‘Mobile Solutions Aiding Knowledge for Health Improvement (M-SAKHI)’ aimed at reducing stunting in infants at 18 months of age.

**Design:**

The PIP was developed using ToC to design the intervention and plan its implementation. Literature review and data from previous pilots helped to identify health service gaps that needed to be addressed by the PIP of this intervention.

**Setting:**

M-SAKHI was implemented in 244 villages under governance of forty primary health centres of Nagpur and Bhandara districts of eastern Maharashtra in central India.

**Participants:**

The study investigators and the public health stakeholders participated in developing the PIP. M-SAKHI evaluation study recruited 2501 pregnant women who were followed up through delivery until their infants were 18 months old.

**Results:**

The PIP was developed, and it identified the following pathways for the final impact: (1) improving maternal and infant nutrition, (2) early recognition of maternal and infant danger signs, (3) improving access and utilisation to healthcare services, (4) improving hygiene, sanitation and immunisation practices, and (5) improving implementation and service delivery of community health workers through their training, monitoring and supervision in real time.

**Conclusion:**

This paper will illustrate the significance of development of PIP for M-SAKHI. It can aid other community-based programmes to design their PIP for nutrition-based BCC interventions.

Childhood stunting affects about 148·1 million children under 5 years of age worldwide, hindering their overall development^(1)^. India accounts for approximately one-third of the world’s total population of stunted children^(2)^. The predominant challenges faced to reduce stunting in children are poor maternal health, inappropriate infant and young child feeding practices^(3,4)^, infections^(5)^, sanitation issues^(5,6)^, poor coverage, delivery, access and utilisation of maternal, neonatal and child health and nutrition services provided by the government. Existing government services struggle to address these challenges^(7,8)^. After the initiation of the National Rural Health Mission in 2005^(8)^, an evaluation of the community health workers, that is, Accredited Social Health Activists (ASHA), was conducted in 2011, and many shortcomings in training and monitoring were identified^(9)^. Therefore, to achieve the desired health impact in the community, there is need for targeted training and effective supervision of ASHA to ensure delivery of community health services and implement interventions.

mHealth interventions that utilise mobile phone technology hold the promise of improving healthcare delivery. Healthcare service coverage can be augmented by reducing outreach time^(10–13)^. A systematic review has shown widespread acceptance of mobile technology in developing countries^(10)^. In India’s healthcare system, separate components of mobile phone technology like apps to aid health workers, text or voice messaging, call centres, etc., have been used. However, it is rare to find an integrated system that incorporates all these separate components in one intervention package^(12–15)^.

Systematic assessments of gaps in the public health systems and knowledge of the cultural and social practices that may affect acceptance of the intervention by the community and the provider are needed to develop an effective intervention. We therefore conducted a series of pilot studies to understand the extent of mobile phone use in the community and women’s acceptance for mHealth intervention during antenatal and postnatal period in Maharashtra. Our first pilot was conducted in 2010 in four urban maternity hospitals, which tested mobile phone-based lactation counselling and health messaging via short text messages (SMS) to promote appropriate infant and young child feeding practices. The results demonstrated that in the mobile-based intervention group, breast-feeding initiation within an hour of birth was significantly higher (36·9 % *v* 23·6 % *P* < 0·001). The rates of exclusive breast-feeding were consistently above 95 % at all follow-up visits. Appropriate complementary feeding was observed in 99·6 % of the intervention group *v* 73·1 % in the control. Infants in the intervention had higher weight than infants in the control group at all follow-up visits. Additionally, 92·3 % of intervention participants expressed satisfaction with the mobile-based breast-feeding counselling^(16)^. Subsequently in a rural community-based qualitative research, we found that women and families were willing to use mobile phones and be contacted for counselling^(17)^. We then assessed the feasibility of using a CommCare app on JAVA phones by ASHA and its implementation challenges^(18)^. The pilot studies helped to design the comprehensive Behaviour Change Communication (BCC) intervention ‘Mobile Solutions Aiding Knowledge for Health Improvement’ or ‘M-SAKHI’ to impact health behaviours of pregnant women and mothers of infants in the selected rural communities to reduce infant stunting and to improve their development.

The description and methodology of evaluation of the effectiveness of M-SAKHI intervention in 2501 pregnant women using cluster-randomised controlled trial to reduce infant stunting was recently published^(19)^. The women were enrolled before 20 weeks of pregnancy and followed up through delivery till their infants were 18 months old. The five components of M-SAKHI intervention were (i) an ‘ASHA’ app for real-time data collection and face-to-face counselling of participants during ASHA’s monthly home visits, (ii) text messages (thrice a week) and voice messages (once a week) sent to the participants by a server, (iii) automated delivery of alert text messages to participants, ASHA and study auxiliary nurse midwife (trained as counsellor), (iv) mobile phone to mobile phone direct counselling of participants every fortnight by study auxiliary nurse midwife counsellor and (v) a field supervision app to monitor ASHA field activities. The outcome indicators, data collection methods and sources are elaborated in Table 1.

**Table 1:** M-SAKHI outcome indicators

Outcome	Description of indicators	Data collection methods and sources
A. Improvement in maternal health outcomes		
Antenatal and postnatal visits	Number of antenatal and postnatal visits at health facility	Separate FRO will collect data from participants on android tablets, using standardised questionnaires, starting at enrolment, once at second and third trimester, at delivery, monthly until postnatal 12 months and then at 15 and 18 months of child’s age
Iron and folic acid (IFA) consumption	Total number of IFA tablets consumed during pregnancy	
Place of delivery	Number of facility and home deliveries	
Mode of delivery	Number of vaginal/C-section deliveries	
Maternal immunisations	Number of tetanus immunisation completed during pregnancy	
Maternal hospitalisations	Number of times mother hospitalised during antenatal and postnatal period	
Maternal mortality rate (MMR)	Number of maternal deaths	
Maternal nutrient intake	Ranking of women’s intake of macronutrient and selected micronutrients assessed by FFQ	Trained FRO will collect data from participants on android tablets, using standard methods of FFQ and Dietary Habits Questionnaire (DHQ) during second or third trimester in pregnancy
Maternal dietary diversity	Consumption of ≥ 4 food groups as assessed by 24-h recall	
Hygiene and sanitation practices	Number of times open defecation	Trained FRO will collect data from participants on android tablets, starting at enrolment, once at second and third trimester, at delivery, monthly until postnatal 12 months and then at 15 and 18 months of child’s age
	Number of times hand washing before meals and after defecation	
	Number of times disposal of stools of infants appropriately	
B. Improvement in fetal/neonatal/infant outcomes		
Fetal loss	Number of miscarriages and abortion	Trained FRO will collect data from participants on android tablets, starting from enrolment, once at second and third trimester and at delivery
	Number of stillbirths	
Preterm deliveries	Number of preterm (based on LMP and EDD < 37 weeks)	
Low birth weight (LBW)	Number of LBW (<2·5 kg) infants	
Neonatal complications	Episodes of birth asphyxia, sepsis, jaundice, pneumonia and diarrhoea and other conditions requiring hospitalisations	Trained FRO will collect data from participants on android tablets, starting from delivery, monthly until 12 months and then at 15 and 18 months of child’s age
Infant immunisations	Number of immunisations completed till 12 months of age	
Infant morbidity	Number of days ill with diarrhoea, fever or cough	
Infant hospitalisations	Number of times neonate/infant hospitalised for any complications	
Rates of neonatal/infant mortality (NMR/IMR)	Number of neonatal and infant deaths	
Exclusive breast-feeding	Proportion of infants 0–5 months of age who are fed exclusively with breast milk	Trained FRO will collect data on android tablets, starting from delivery, monthly until 12 months and then at 15 and 18 months of child’s age based on WHO 24-h recall method.
Early initiation of breast-feeding	Proportion of children born in the last 24 months who were put to the breast within 1 hour of birth	
Timely introduction of solid, semi-solid or soft foods	Proportion of infants 6–8 months receiving solid, semi-solid or soft foods	
Minimum dietary diversity	Proportion of children 6–23 months who receive foods from 4 or more food groups	
Minimum meal frequency	Proportion of breastfed and non-breastfed children aged 6–23 months, who receive solid, semi-solid or soft foods (but also milk feeds for non-breastfed children) the minimum number of times or more.	
Stunting or low length-for-age and wasting or low weight for length	The proportion of children at 2,4, 6, 8, 10, 12, 15 and 18 months with low length-for-age or weight-for-height *Z* score (< –2 *Z* calculated from the 2006 WHO growth standard).	Trained AFRO will collect data on android tablets using CommCare app. For anthropometry every second month from birth until 12 months and then at 15 and 18 months. For infant diet at 10, 12, 15 and 18 months
Nutrient adequacy of infant diet	Quantified macro- and micronutrient intakes from dietary recalls and comparisons to national/WHO recommended nutrient intakes	
Infant development assessed by Ages and Stages Questionnaire 3rd edition – ASQ 3	ASQ scores to assess the motor (fine and gross), language (receptive and expressive) and cognitive development of infant	Trained AFRO will collect data using ASQ 3 scoring paper forms, once at 12 and 18 months
C. Other outcomes		
Knowledge and performance assessment of ASHA	Number of ASHA with adequate grades in pre- and post-training knowledge tests. Proportion of ASHA receiving with good participant visit targets (those receiving green icons in FS app)	These scores will be obtained by ASHA in pre- and post-test and their performance as assessed by project managers.^*^
Cost-effectiveness	Cost of developing and implementing M-SAKHI intervention Incremental cost-effectiveness ratio of the M-SAKHI intervention compared to usual care Cost per child of underweight saved by the intervention	Trained FRO will collect data in both arms from participants on standardised cost paper forms, during monthly home visits, starting from enrolment until child is 18 months of age

M-SAKHI, Mobile Solutions Aiding Knowledge for Health Improvement; FRO, field research officers; AFRO, field research officers specially trained to collect anthropometry, child development and dietary data; FS, field supervisor; ASHA, Accredited Social Health Activists.

* All data collection will be collected for both intervention and control arms, except the knowledge and performance of ASHA which will be conducted only in the intervention arms.

**SOURCE:** Patel AB, Kuhite PN, Alam A, *et al.* M-SAKHI – mobile health solutions to help community providers promote maternal and infant nutrition and health using a community-based cluster randomised controlled trial in rural India: A study protocol. *Matern Child Nutr*. 2019; e12850. https://doi.org/10.1111/mcn.12850.

In this manuscript, we explain the Program Impact Pathway (PIP) for the development of M-SAKHI using Theory of Change (ToC) approach and the methods for Process Evaluation. The ToC provides a structured method for mapping cause-and-effect relationships in social interventions, illuminating how specific activities drive desired outcomes^(20,21)^. The ToC approach identifies pivotal triggers for behavioural change and enables meticulous planning and evaluation of interventions. The PIP frameworks play a vital role in providing a structured framework for planning, monitoring and evaluating the implementation of complex interventions. The PIP ensures that activities are carried out as intended and helps to track the progress of the intervention. It helps to provide informed decisions for improvements in future implementations^(22)^. The Process Evaluation forms an integral part of any PIP analysis. It helps to identify which components of the intervention are likely to work, what will not work and which areas need to be strengthened^(23,24)^. BCC interventions involving mHealth are often resource-intensive and need continuous monitoring and evaluation to be successful. Publications describing the PIP for complex BCC interventions are scarce^(25)^. The description of how the impact pathways are developed may contribute towards designing impact assessments of similar interventions.

## Methods

The detailed description of how we developed the PIP using the ToC approach for M-SAKHI are provided below.

### Step 1: identifying gaps in the health systems and strategies

We conducted a systematic review of literature and searched electronic bibliographic databases like PubMed, Embase, Web of Science and Scopus using key words like nutrition/ health/ mHealth intervention /rural India /ASHAs /ASHA evaluation /ASHA workload/gaps in service delivery, etc., to retrieve publications^(7,9,26–31)^. Additionally, the results from the series of pilot studies helped assess the cultural and social practices that may affect acceptance of the intervention by the community and the provider^(16–18)^. Table 2 enlists the major gaps in health system service delivery and factors responsible for infant stunting and how we planned to address these gaps through the M-SAKHI intervention.

**Table 2:** Gaps in existing health system and factors responsible for stunting (identified through literature review) and strategies to address these using M-SAKHI intervention

S. NO.	Identified gaps/problems	Solutions/pathways	M-SAKHI intervention strategy to address these gaps
1.	Low maternal, neonatal and child health services coverage by ASHA^(7–9,26–30)^	Improving coverage of maternal and infant care services	i) A CommCare – ASHA app which will be user-friendly and standardise the data collection methods to improve motivation and involvement of ASHA.
2.	Poor healthcare service delivery by ASHA and low care seeking by communities for complicated cases^(7,9,28–30)^	Improving ASHA knowledge and skills and Improving health awareness in participants regarding early identification of illnesses/danger signs	App embedded coloured images, audio-video counselling messages in the CommCare – ASHA app for early identification of danger signs by the ASHA and the participant.
			Server delivered alerts in the form of text messages to be sent to: participants for reinforcing the seriousness of complications and the ASHA for facilitating referrals
			Cell phone counselling by study auxiliary nurse midwife counsellor to provide counselling to received alert notifications (alert messages delivered to study auxiliary nurse midwife counsellor, ASHA and the participants).
3.	Inadequate supervision and support to ASHA^(7)^	Improving monitoring and supervision systems for ASHA	A separate CommCare – field supervisory app that enables the ASHA supervisors to monitor and grade the ASHA performance and identify weaker ASHA for timely re-trainings
4.	Poor maternal health and nutrition^(3)^	Improving awareness in participants and care providers (ASHA) regarding: Nutrition during pregnancy and lactation Early identification of antenatal and postnatal complications Utilisation of healthcare services – antenatal and postnatal visits, IFA consumption Immunisations and – timely reporting to healthcare facility	ASHA app with audio-visual aids to enhance face-to-face counselling Cell phone counselling by study auxiliary nurse midwife counsellor Server delivered text and voice messages
5	Inadequate adoption of infant and young child feeding practices^(4)^	Improving awareness regarding infant feeding practices	ASHA app with audio-visual aids to enhance face-to-face counselling Cell phone counselling by study auxiliary nurse midwife counsellor
6	Poor hygiene and sanitation practices^(5,6)^	Improving health awareness in participants for appropriate hygiene and sanitation practices	ASHA app with audio-visual aids to enhance face-to-face counselling Server delivered alerts and text/voice messages

M-SAKHI, Mobile Solutions Aiding Knowledge for Health Improvement; ASHA, Accredited Social Health Activists; IFA, iron and folic acid.

### Step 2: developing the Theory of Change model for the M-SAKHI intervention

The ToC model or the pathway to change was developed by ‘backward mapping’ from the desired goal of achieving reduction in rates of infant stunting^(32,33)^. The pathway includes the outcomes needed to achieve the goal (of reducing stunting), the change in behaviour (the outputs) that enables the desired outcomes, the contextual factors at individual, societal and system level that need to be considered for attaining these outcomes, and the processes that need to be adopted to obtain the identified solutions (Table 3). The M-SAKHI outcomes were specific, reliable, measurable and time-bound indicators that are described in Table 1. The contextual factors at the individual and societal level were the extent of health awareness of the participant and cultural acceptance of the intervention by the family and community. At the health system level, barriers and facilitators for implementation were identified. It was assumed that if these contextual factors are addressed, then there will be an acceptable level of participation of the individual, the family and the ASHA^(34–36)^. The processes and inputs were the five components of the intervention and the resources required for developing and implementing the intervention respectively.

**Table 3:** The Theory of Change hypothesising behaviour change in communities through M-SAKHI intervention

	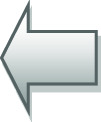	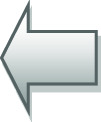	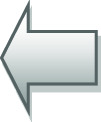	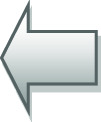	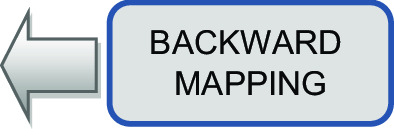	
Problem	Solution	Processes	Assumptions/contextual factors	Changed behaviour	Outcomes	Impact
Lack of awareness regarding maternal nutrition	Promoting awareness and improving knowledge regarding appropriate nutrition during pregnancy and lactation	Data collection in real time on mobile phones and face-to-face counselling using ASHA app every month Server delivered text messages sent to participants thrice a week and server delivered voice messages sent to participants once a week Server delivered alerts (text messages) regarding danger signs sent to participant, ASHA and by study auxiliary nurse midwife counsellor Mobile phone to mobile phone counselling by a study auxiliary nurse midwife counsellor to participant every fortnight Field supervisory app for real-time monitoring of community health workers	Social context of the intervention programme sites Barriers to implementing and delivering the programme Completeness of the programme Retaining the new skills gained through the programme Engagement of the participants and their families as well as community health workers in the programme Extent of reach of the programme Dose delivered of the programme Barriers and facilitators to adherence to the messages delivered	Taking appropriate care and nutrition during pregnancy and lactation	Changes in maternal nutrient intakes and dietary diversity leading to improved maternal nutrition	Reduction in STUNTING Improved INFANT DEVELOPMENT
Lack of awareness regarding infant and young child feeding practices	Promoting awareness and improving knowledge regarding appropriate infant and young child feeding practices			Adopting adequate infant and young child feeding practices	Changes in rates of exclusive breast-feeding, early initiation of breast-feeding, duration of breast-feeding, and timely introduction of solids and semi-solids. Minimum dietary diversity, minimum meal Frequency and nutrient adequacy of infant diet leading to improved infant and young child nutrition	
Lack of awareness regarding complications during antenatal and postnatal period in mother and child	Promoting awareness and improving knowledge regarding early identification of danger signs/illnesses			Early recognition of illnesses/danger signs	Changes in the rates of maternal and infant complications leading to reduced maternal and infant morbidities	
Poor access and utilisation of healthcare services	Promoting awareness and improving knowledge regarding antenatal and postnatal visits, regular check-ups, facility deliveries, IFA consumption, immunisations, early care seeking at health facility, etc.			Completing antenatal and postnatal visits, regular check-ups at health facilities, birth preparedness and tracking infant anthropometry	Changes in antenatal and postnatal visits, IFA consumption, Hb levels, facility deliveries, maternal and infant immunisations, infant growth indices leading to improved antenatal and postnatal care services	
Lack of awareness regarding hygiene and sanitation practices	Promoting awareness and improving knowledge regarding adopting correct WASH practices			Adopting correct hygiene and sanitation practices	Improved hygiene and sanitation practices	
Lack of adequate monitoring and supervision of community health workers	Developing systems and improving monitoring and supervision of community health workers			Improving motivation and involvement of community health workers	Changes in knowledge and skills of community health workers, thus improving efficiency and service delivery	

M-SAKHI, Mobile Solutions Aiding Knowledge for Health Improvement; ASHA, Accredited Social Health Activists; IFA, iron and folic acid.

### Step 3: consultative process with the stakeholders for designing the Program Impact Pathway

The preliminary framework for the PIP was conceptualised before the start of programme by the study team at Lata Medical Research Foundation and University of Sydney. The framework was developed by using lessons from the previous pilot studies^(16–18)^, consultations with international experts in PIP, dialogue with key stakeholders, implementers and the recipients of the programme. The stakeholders were private mobile phone companies, the technology providers, public health systems, public health academicians and experts and the donor organisations. This draft was then refined with the help of programme implementation experts at the Nutrition Embedding Evaluation Program – of PATH (global health organisation), global meeting held in Barcelona, Spain^(37)^. Components of the programme contributing towards the goal were enumerated, and their pathways to achieve the impact were identified. The intervention components were further refined contextually in the consultative process, based on community-based qualitative research. A Process Evaluation plan aligning with the PIP was developed to evaluate the impact of the M-SAKHI intervention.

## Results

### The M-SAKHI Program Impact Pathway framework

**Fig. 1:**
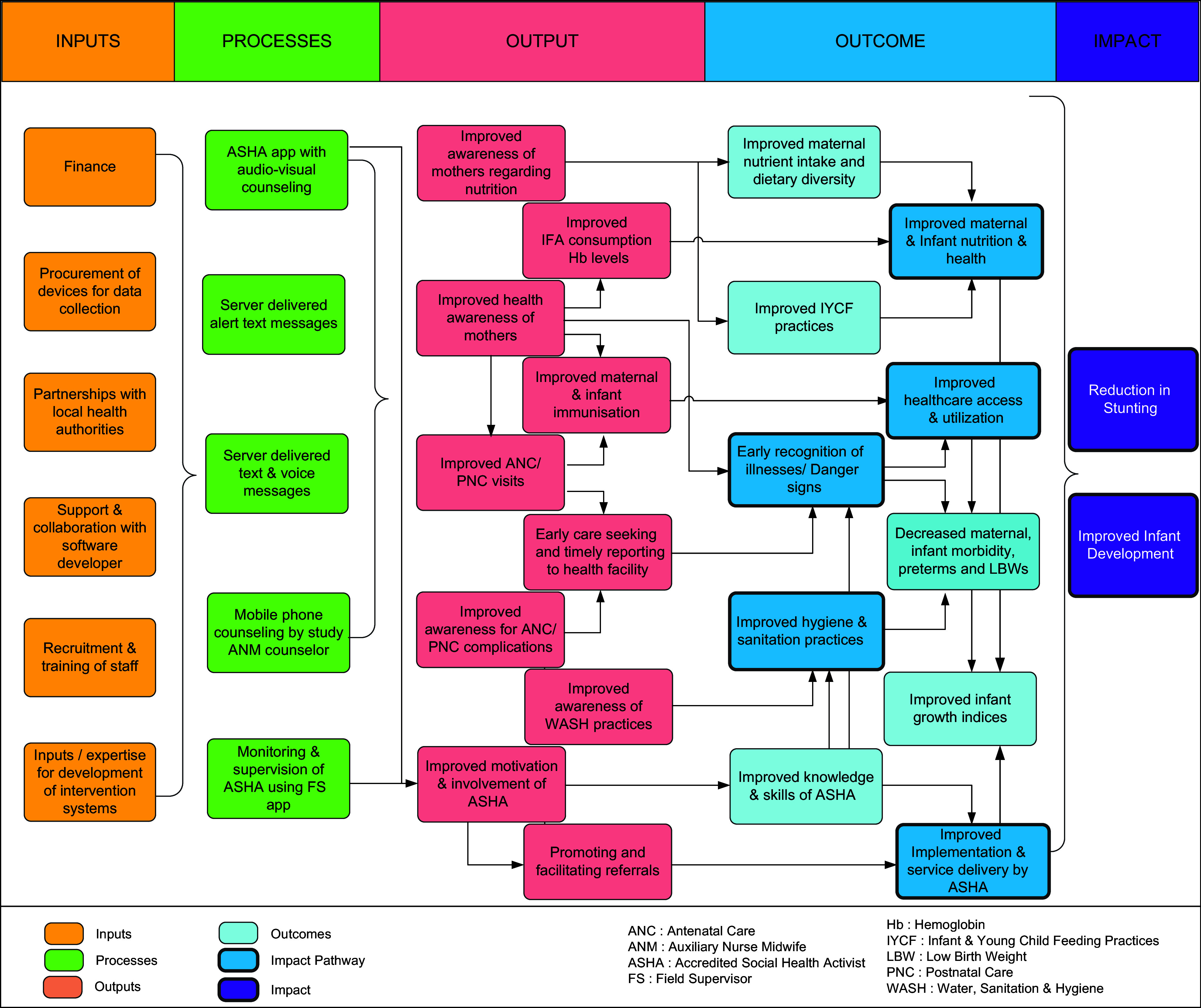
M-SAKHI Program Impact Pathway framework. M-SAKHI, Mobile Solutions Aiding Knowledge for Health Improvement

This framework (Fig. 1) was developed using the steps mentioned above. The ‘Inputs’ included resources needed to set up the systems for the intervention. The ‘Processes’ were the activities that were enabled by the intervention such as ASHA app for face-to-face counselling, server delivered text messages, voice messages, automated delivery of alert text messages and mobile phone counselling by study auxiliary nurse midwife counsellor. These processes are expected to achieve the ‘Outputs’, that is, improved awareness of mothers regarding nutrition, water, hygiene and sanitation, improved awareness regarding her health, and improved maternal and infant immunisations. The outputs for the community health workers included improved awareness and motivation of ASHA to help mothers adopt healthy behaviour and facilitate timely referrals to healthcare centres when needed. The improved awareness regarding health behaviour and nutrition is expected to result in ‘Outcomes’, that is, improvement in practices pertaining to maternal nutrition and maternal weight; improved infant and young child feeding practices and thus improved infant weight; improved hygiene and sanitation practices; early recognition of illnesses/danger signs; improved health-seeking behaviour and utilisation of health services (antenatal and postnatal care); decreased maternal and infant morbidities; and decreased preterm births and low birth weight babies. For the community health workers, the outcomes were improved knowledge and skills and improved implementation and service delivery by ASHA.

As shown in Fig. 1, the ‘Inputs, Processes, Output and Outcomes’ are expected to result in the final ‘Impact’ of reducing stunting and improving development of infants at the age of 18 months through five pathways (represented by dark blue coloured boxes in Fig.1). The pathways are (1) improving maternal and infant nutrition, (2) early recognition of maternal and infant danger signs, (3) improving access and utilisation to healthcare services, (4) improving hygiene, sanitation and immunisation practices and (5) improving implementation and service delivery of community health workers through their training, monitoring and supervision in real time.

### The Process Evaluation plan

This plan is to evaluate the following seven attributes of the M-SAKHI intervention: (i) fidelity – this is the extent to which the intervention will be implemented as planned. (ii) Dose delivered – this will be evaluated by assessing the number of activities/deliverables. (iii) Dose received (exposure) – this will be evaluated by assessing the extent to which participants actively engage with, interact with and/or use M-SAKHI programme components and activities. (iv) Dose received (satisfaction) – this will be evaluated by assessing the participants’ satisfaction with the programme and the interaction with those who deliver it. (v) Reach – this will be assessed by proportion of target group that participated in the programme. (vi) Recruitment – this will be assessed using methods of communicating and recruiting participants, and maintaining their participation. (vii) Contextual factors will consider the elements of the physical, social, cultural and political environment that influence implementation or outcomes. All seven attributes will be assessed for each of the implementation channels of the intervention, namely – use of ASHA app for face-to-face counselling, phone counselling by study auxiliary nurse midwife counsellor, text, alert messages and voice messages and the field supervisory app. The data sources to be used will be qualitative based on interviews or quantitative based on data regarding field implementation activities, that is, available on the server.

## Discussion

Although there are studies analyzing the PIP frameworks, literature describing the development process of the PIP is sparse^(38,39)^. Through this paper we provide information on how a PIP was developed for a mHealth BCC intervention – M-SAKHI to address gaps in the current healthcare delivery systems. The M-SAKHI intervention was provided to pregnant mothers, enrolled before 20 weeks of gestation, through their delivery till their infant was 12 months old with the goal to reduce infant stunting and improve infant development measured at 18 months of age. The M-SAKHI intervention is intended to be user-friendly, culturally appropriate and consistent with the current popularity of use of mobile phones for communication. It focuses on knowledge and awareness components to improve health and nutrition, both at the level of uptake by the participant and delivery by the ASHA. On the delivery side, it intends to improve service delivery by enhancing knowledge, skills and efficiency of rural community health workers by use of technology^(40)^. It has been observed that adoption of appropriate maternal, neonatal and child healthcare practices are dependent on how the healthcare providers communicate and influence their clients to adopt healthy behaviour change practices^(41)^. Therefore, currently different BCC methods are being used to improve maternal, neonatal and child health practices, including nutrition in multiple settings. Community-based BCC programmes often have ambitious aims and are challenging to implement. Thus, they mandate effective planning, assessment of programme feasibility, identification of the needs of the end user and proper mapping of the all steps in order to have the desired impact. Despite an effective design, due to implementation challenges many programmes fail to assess what worked well and what did not in their programme. A well-described PIP and the Process Evaluation plan helps to evaluate and monitor the implementation process^(42)^. Using the ToC approach (Table 3), we developed the PIP (Fig. 1) and the Process Evaluation plan for the M-SAKHI intervention.

ToC approach facilitates the designing of the intervention and the PIP framework^(43,44)^. It provides a complete framework for understanding why the programme is needed, what will be its impact, how the programme will create its value in community and how it can use data to improve its impact in the future^(45)^. A strong ToC model reveals the hidden assumptions and challenges from people in different roles, levels and perspectives within the programme, facilitating agreement between them and negotiating shared commitment among them^(46)^. Thus, it will make the programme easier to replicate, sustainable, scalable and evaluate as it defines each of the necessary steps within the theory.

The M-SAKHI PIP describes the inputs, the processes leading to the proposed outputs and outcomes and the final impact of achieving reduction in stunting and improving infant development. We expect that this PIP framework will not only help monitor and resolve the key challenges that are necessary for smooth implementation of M-SAKHI but also aid in identifying opportunities for improving the impact of the programme. It will generate evidence on how the intervention is expected to impact the community health worker’s skills, knowledge, performance, the participants’ knowledge, the utilisation of the intervention by the participants and the desired transformation in health-related behaviours to achieve health goals. The Process Evaluation includes assessment of facilitators and barriers through interviews with key stakeholders which include programme administrators, programme managers, front-line workers and programme clients. The information is used to monitor the programme through feedback mechanisms ensuring desirable intervention outcomes^(23,35,47–49)^. This process helps service providers and utilisers to assess the reliability of the intervention, the training processes, the quality and acceptability of the delivered outcomes for replicability, scalability and sustainability. The results from this trial will provide lessons and map steps for successful implementation of similar large-scale programmes. It can help development of frameworks for other similar community-based programmes to improve their design, delivery, utilisation and sustainability.

## Conclusion

The PIP using the ToC approach was formulated to plan the development of essential components of the M-SAKHI intervention and to enable mapping, monitoring and evaluation of the intervention implementation activities. We provide a comprehensive description of how the PIP was prepared for M-SAKHI, a mHealth-based BCC intervention for rural pregnant women implemented by community health workers and auxiliary nurse midwives. This description aims to establish the significance of a PIP that enables implementation of the intervention that is intended to achieve improvement in childhood nutrition and combat infant stunting in rural communities.
